# Tumor necrosis factor α release in peripheral blood mononuclear cells of cutaneous lupus and dermatomyositis patients

**DOI:** 10.1186/ar3549

**Published:** 2012-01-04

**Authors:** Adam S Nabatian, Muhammad M Bashir, Maria Wysocka, Meena Sharma, Victoria P Werth

**Affiliations:** 1 Philadelphia Veterans Affairs Medical Center, 38th and Woodland Avenues, Philadelphia, PA 08901, USA; 2 Department of Dermatology, School of Medicine, University of Pennsylvania, 2 Maloney Building, 36th and Spruce Streets, Philadelphia, PA 19104, USA; 3 University of Medicine and Dentistry of New Jersey-Robert Wood Johnson Medical School, 675 Hoes Lane West Piscataway, New Brunswick, NJ 08854 USA

## Abstract

**Introduction:**

Several studies have reported that TNFα is substantially increased within skin lesions of patients with discoid lupus erythematosus (DLE), subacute cutaneous lupus erythematosus (SCLE) and dermatomyositis (DM) compared to controls. Elevated TNFα has been reported in the sera of some patients with systemic lupus erythematosus, DLE and SCLE, but not in the sera of patients with DM. Because of the key pathogenic role of autoimmunity in these diseases, in this study we sought to evaluate TNFα production by a readily available source of immune cells (namely, peripheral blood mononuclear cells (PBMCs)) taken from controls and from patients with cutaneous lupus or DM.

**Methods:**

Freshly isolated PBMCs were cultured overnight, and TNFα protein accumulation in conditioned medium was determined. In addition, flow cytometry using cell-type-specific markers was performed to determine the sources of TNFα. One-way analysis of variance and Dunnett's multiple comparisons test were performed for statistical comparisons.

**Results:**

Accumulation of TNFα protein in conditioned medium containing PBMCs from DLE patients, but not from SCLE, TLE or DM patients, was significantly greater (19-fold) than that from controls (*P *< 0.001). In DLE PBMCs, increased TNFα was produced by circulating monocytes and myeloid dendritic cells (mDCs). The mean TNFα fluorescence intensity, but not the total number, of both monocytes and mDCs (*P *< 0.01) from DLE patients was significantly greater (2.3-fold) than that of controls. There were significantly more (13.3-fold) mDCs with intracellular TNFα in blood from DLE patients (*P *< 0.001) and DM patients (*P *< 0.001) compared to controls. Most importantly, a positive correlation was seen in DLE patients between their disease activity measured using the Cutaneous Lupus Erythematosus Disease Area and Severity Index and TNFα protein secretion (*r *= 0.61, *P *< 0.08).

**Conclusions:**

TNFα protein production by PBMCs is greater in DLE patients than in patients with other cutaneous forms of lupus and DM or in controls. Flow cytometric studies demonstrated that circulating monocytes and mDCs contributed to this increased TNFα production. Monocytes and mDCs are present in lesional skin, and the increased TNFα production by these cells and other PBMCs likely increase the number of inflammatory cells seen in DLE skin relative to other subsets of cutaneous lupus erythematosus and DM. These results provide a possible biological explanation for the denser infiltrate seen in DLE relative to DM.

## Introduction

Lupus erythematosus (LE) is a chronic autoimmune inflammatory disease. Skin involvement occurs in 70% to 85% of all patients with LE [1]. Cutaneous disease is classified as LE-specific or LE-nonspecific, based on assessments of the morphology of the cutaneous lesions and the results of histopathologic examinations [2]. LE-specific skin lesions are divided into three broad categories that include chronic cutaneous lupus erythematosus (CCLE), subacute cutaneous lupus erythematosus (SCLE) and acute cutaneous lupus erythematosus [3]. Herein we focus on two subsets of CCLE: (1) discoid lupus erythematosus (DLE) and tumid lupus erythematosus (TLE) and (2) SCLE. DLE typically presents as scaly, erythematous, disk-shaped patches and plaques, whereas TLE manifests as single or multiple raised polycyclic erythematous plaques with a bright reddish or violaceous smooth surface that does not scar [1]. SCLE typically presents as erythematous papulosquamous, psoriasiform plaques or annular-polycyclic plaques and the lesions typically resolve without scarring [4]. Dermatomyositis (DM) is a chronic inflammatory disorder of the skin and muscles, with histologic findings similar to SCLE [5,6].

TNFα is a critical proinflammatory cytokine that is implicated in the pathogenesis of multiple inflammatory diseases. TNFα can be produced by many different cell types including monocytes, macrophages, dendritic cells, T and B lymphocytes, natural killer cells, neutrophils, mast cells, endothelial cells, fibroblasts and keratinocytes [7-9]. TNFα is produced as pro-TNFα, which is expressed on the plasma membrane, where it can be cleaved in the extracellular domain by ADAM17 (also known as "TACE," or TNFα-converting enzyme), which is a matrix metalloproteinase, and results in the release of TNFα in a soluble form [10]. ADAM17 mRNA expression was found to be increased in lesional skin from psoriasis patients [11]. Both membrane-associated and soluble TNFα are active [5].

Several studies have examined TNFα in skin lesions and serum of patients with CLE and DM. TNFα is increased in lesional skin of patients with DLE and SCLE compared to controls [12-15]. DM lesional skin expresses TNFα, but staining has been found to be more evident in DLE patients and absent in control specimens [5]. TNFα was increased in muscle biopsies of DM patients [16]. TNFα has been reported to be elevated in sera of some patients with systemic lupus erythematosus (SLE), DLE and SCLE, but not in that of patients with DM [17-20]. *In vitro *production of TNFα by unstimulated PBMCs from juvenile DM patients was measured in a previous study in which the investigators found that PBMCs from children who had a disease course ≥ 36 months produced more TNFα compared to PBMCs from juvenile DM patients who had had the disease < 36 months [21]. As there are data regarding TNFα expression in the skin and sera of CLE and DM patients, but, to the best of our knowledge, minimal data regarding TNFα production from the PBMCs of these patients, the aim of our study was to evaluate the production of TNFα by PBMCs from DLE, SCLE, TLE and DM patients.

## Materials and methods

### Subjects

Twenty-one DLE, ten SCLE, ten TLE and eighteen DM patients were recruited from the outpatient autoimmunity clinic in the Department of Dermatology at the Hospital of the University of Pennsylvania. All subjects who had the diagnosis of DLE, SCLE, TLE and/or DM were invited to participate in the study. Seven of the DLE patients and one of the SCLE patients had SLE as well. All of the recruited patients were receiving therapy to treat their disease (see Table 1). Eighteen people without LE, DM or any autoimmune disease were recruited as controls. To be eligible for the study, controls must not have been on corticosteroids or immunosuppressive drugs.

**Table 1:** Patient demographics and clinical characteristics^a^

Disease	Sex (*n*)	Race (*n*)	Mean age, years (range)	Mean CLASI or CDASI score	Medications (patients, *n*)
DLE (*n *= 21)	Females, 16 Males, 5	AA, 12 White, 8 Hispanic, 1	45.0 (23 to 64)	8	Antimalarials, 18 Methotrexate, 2 Immunosuppressants, 5 Corticosteroids, 3 Dapsone, 1 Thalidomide, 1
SCLE (*n *= 10)	Females, 7 Males, 3	White, 9 Asian, 1	47.1 (20 to 64)	9.5	Antimalarials, 7 Immunosuppressants, 2 Corticosteroids, 2 Thalidomide, 4
TLE (*n *= 10)	Females, 8 Males, 2	AA, 1 White, 9	42.9 (25 to 57)	10.1	Antimalarials, 10 Methotrexate, 1
DM (*n *= 18)	Females, 15 Males, 3	AA, 1 White, 16 Asian, 1	49 (21 to 75)	14.7	Antimalarials, 10 Methotrexate, 6 Immunosuppressants, 6 Corticosteroids, 9
controls (*n *= 18)	Females, 10 Males, 8	AA, 4 White, 12 Asian, 2	31.2 (26 to 57)	N/A	N/A

^a^AA = African American; CDASI = Cutaneous Dermatomyositis Area and Severity Index; CLASI = Cutaneous Lupus Erythematosus Disease Area and Severity Index; DLE = discoid lupus erythematosus; DM = dermatomyositis; N/A = not applicable; SCLE = subacute cutaneous lupus erythematosus; TLE = tumid lupus erythematosus.

Demographic information and data regarding current medications and comorbid illnesses were collected from all subjects. The treating dermatologist evaluated each LE and DM patient by performing a complete physical examination and assessing the patient based on Cutaneous Lupus Erythematosus Disease Area and Severity Index (CLASI) and Systemic Lupus Erythematosus Disease Activity Index (SLEDAI) scores for LE patients and the Cutaneous Dermatomyositis Area and Severity Index (CDASI) scores for DM patients to measure disease activity and damage [22,23]. This study was approved by the Institutional Review Board (IRB) at our institution, and all PBMCs were obtained according to the IRB protocol. The Declaration of Helsinki protocols were followed, and patients gave their written, informed consent.

### Cell culture and TNFα determination by ELISA

PBMCs were separated from heparinized venous blood of patients with DLE, SCLE, TLE and DM, as well as from controls, on an endotoxin-free Ficoll-Paque PLUS™ gradient (GE Healthcare Bio-Sciences AB, Uppsala, Sweden). PBMCs were cultured overnight in RPMI 1640 medium supplemented with 10% fetal bovine serum, 1% L-glutamine and 1% penicillin-streptomycin at 37°C and 5% CO_2_. The following morning PBMCs (1 × 10^5 ^cells in 0.2 ml per well) were plated in 96-well plates (Falcon, Franklin Lakes, NJ, USA) and cultured for 24 hours at 37°C and 5% CO_2_. Conditioned media were then collected and stored at -80°C. TNFα was quantitated by ELISA according to the manufacturer's protocol (BD Biosciences, San Diego, CA, USA). All determinations were performed in triplicate. The viability of cells was measured by trypan blue staining.

### Flow cytometric analysis

Prior to intracellular staining for TNFα, PBMCs were cultured in medium for 4 hours in the presence of brefeldin A (1 μg/ml; Invitrogen/Life Technologies, Carlsbad, CA, USA). To detect intracellular expression of TNFα in dendritic cells, approximately 10^6 ^PBMCs per sample were stained with lineage 1 cocktail-fluorescein isothiocyanate (FITC) (lineage cocktail containing antibodies against CD3, CD14, CD16, CD19, CD20 and CD56), anti-human leukocyte antigen (HLA)-DR-perdinin-chlorophyll-protein (PerCp) and anti-CD123-phycoerythrin (PE) (plasmacytoid dendritic cells (pDCs)) or anti-CD11c-PE (myeloid dendritic cells (mDCs)) for 30 minutes on ice, followed by 15-minute incubation with fixation reagent A (FIX & PERM; Invitrogen/Life Technologies). Cells were then washed, permeabilized with reagent B (FIX & PERM; Invitrogen Life/Technologies) and stained with anti-TNFα-allophycocyanin (anti-TNFα-APC) antibody (or an appropriate isotype control) for 15 minutes at room temperature.

To detect intracellular expression of TNFα in monocytes, PBMCs were stained with anti-CD64-FITC and anti-CD14-PE followed by anti-TNFα-APC or an appropriate isotype control as described above. To detect intracellular expression of TNFα in T cells, PBMCs were stained with anti-CD3-FITC followed by anti-TNFα-APC or an appropriate isotype control as described above. All antibodies used were purchased from BD Biosciences.

Cells were analyzed using a FACSCalibur flow cytometer (BD Biosciences, San Jose, CA, USA) with BD CellQuest software (BD Biosciences) at the Flow Cytometry and Cell Sorting Core, Abramson Cancer Center, University of Pennsylvania, Philadelphia, PA, USA. We acquired 150,000 events of live cells.

### Statistical analysis

One-way analysis of variance followed by Dunnett's multiple comparisons test was used for statistical evaluation as appropriate. *P *values less than 0.05 in the *post hoc *test were considered significant. Correlations were calculated by performing a Pearson correlation test.

## Results

### Study population

The demographic and clinical characteristics of the study population are summarized in Table 1. Seven of the DLE patients and one of the SCLE patients had SLE, which did not affect the study results, because removal of these patients from their respective groups had no effect on mean values or statistical significance. All of the recruited patients were receiving therapy to treat their disease; however, there was no correlation between the therapy that the patients were receiving and the results obtained.

### TNFα release from unstimulated DLE PBMCs was significantly elevated and correlated with disease activity

**Figure 1:**
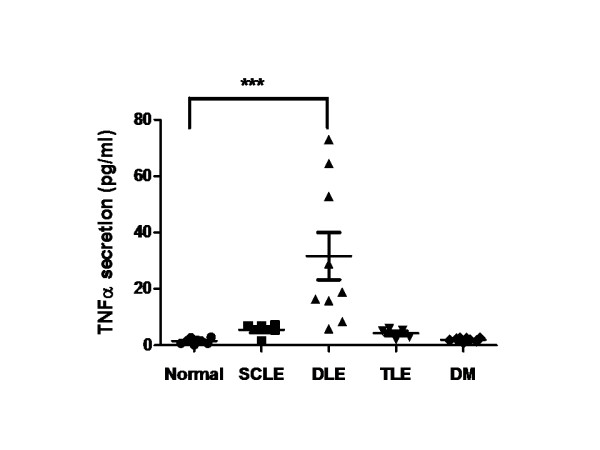
**TNFα protein production by unstimulated peripheral blood mononuclear cells from DLE, SCLE, tumid TLE and DM patients and from controls**. TLE should be written in the figure Peripheral blood mononuclear cells (PBMCs) were cultured for 24 hours, followed by collection of supernatants. The concentration of TNFα in the supernatants was measured by ELISA. Bars show the means and SD. ***P < 0.001 vs control PBMCs by Dunnett's multiple comparison test. DLE = discoid lupus erythematosus, DM = dermatomyositis, TLE, tumid lupus erythematosus, SCLE, subacute cutaneous lupus erythematosus.

**Figure 2:**
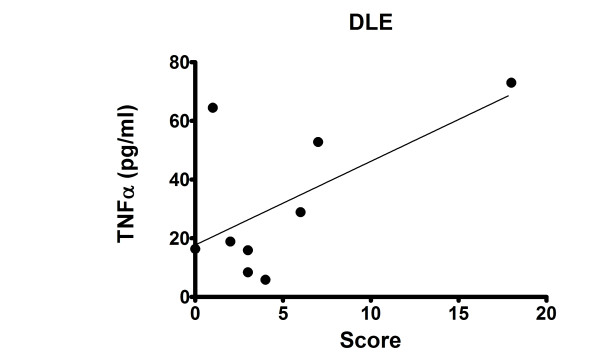
**TNFα in peripheral blood mononuclear cell supernatants from discoid lupus erythematosus (DLE) patients correlated with Cutaneous Lupus Erythematosus Disease Area and Severity Index scores (*r *= 0.61)**.

Initially, we compared the amount of TNFα released from PBMCs of nine DLE, five SCLE, five TLE and eight DM patients, as well as eight controls, by ELISA. Compared to controls, DLE PBMCs produced significantly greater amounts of TNFα (*P *< 0.001) (Figure 1). However, SCLE, TLE and DM PBMCs did not produce significantly greater amounts of TNFα compared to controls. The variation of the triplicate of TNFα levels for each patient was < 10%. The production of TNFα was not induced by lipopolysaccharide (LPS) contamination, because the maximal level of LPS detected by Limulus amoebocyte assay (Associates of Cape Cod, East Falmouth, MA, USA) was < 0.015 EU/ml (< 1.5 pg/ml), a concentration which gives a negative test result. We also determined the correlation of TNFα release with disease activity in these nine DLE patients using the CLASI score to measure disease activity. A positive correlation (*r *= 0.61, *P *= 0.08) was seen in DLE patients between their disease activity and TNFα protein secretion (Figure 2). There was no correlation seen in SCLE (*r *= -0.05) or DM (*r *= 0.3493) patients between their disease activity and TNFα protein secretion.

### Myeloid dendritic cells expressing intracellular TNFα were significantly greater in DLE and DM patients than in controls

**Figure 3:**
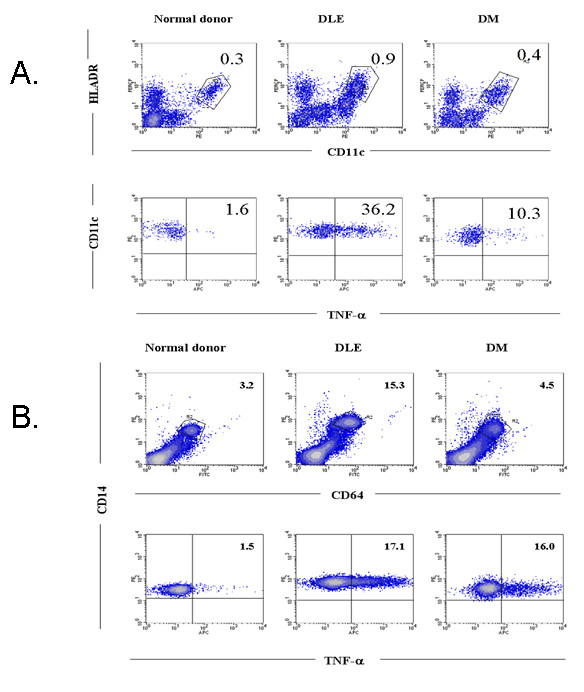
**Flow cytometric analysis of intracellular TNFα production by myeloid dendritic cells and monocytes from patients and controls**. Upper panels show gating of peripheral blood mononuclear cells (PBMCs) from representative patients with discoid lupus erythematosus (DLE), dermatomyositis (DM) or controls stained with fluorochrome-conjugated appropriate antibodies **(A) **myeloid dendritic cells (lineage negative, HLADR^+^, CD11c^+^) or **(B) **monocytes (CD64^+^CD14^+^). Lower panels show intracellular expression of TNFα in **(A) **myeloid dendritic cells or **(B) **monocytes. The numbers in the upper panels state the percentage of cell subset per total population of PBMCs. The numbers in the lower panels state the percentage of myeloid dendritic cells or monocytes producing TNFα.

The dot plots shown in Figure 3 demonstrate populations of both mDCs (Figure 3A) and monocytes (Figure 3B) from representative individuals, one from each group, who were defined and/or gated by antibodies (upper panel) and subsequently analyzed for their ability to produce TNFα (lower panel).

**Figure 4:**
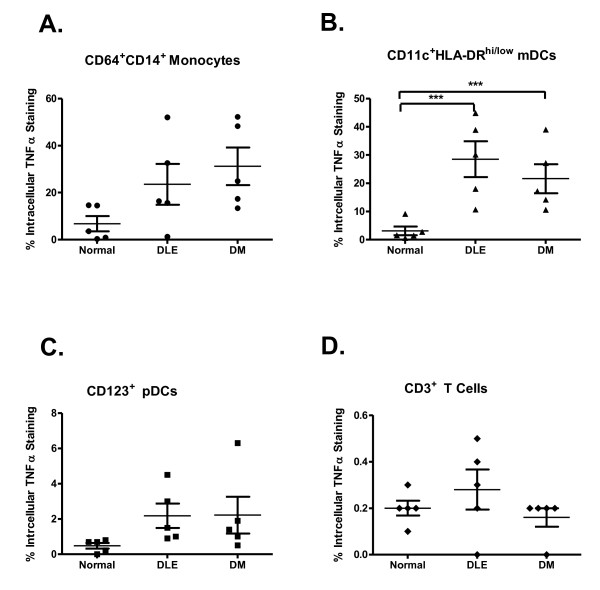
**Defining the cell population responsible for TNFα production**. **(A) **CD64^+^CD14^+ ^monocytes, **(B) **CD11c^+^HLA-DR^hi/low ^myeloid dendritic cells, **(C) **CD123^+ ^plasmacytoid dendritic cells and **(D) **CD3^+ ^T lymphocytes from five discoid lupus erythematosus (DLE) and five dermatomyositis (DM) patients, as well as from five controls, were analyzed by flow cytometry to assess their ability to produce intracellular TNFα. Bars show the means and SD. *** *P *< 0.001 vs controls by Dunnett's multiple comparisons test.

We determined the percentage of CD64^+^CD14^+ ^monocytes, CD123^+ ^pDCs, CD11c^+^HLA-DR^hi/low ^mDCs and CD3^+ ^T lymphocytes expressing intracellular TNFα in five DLE, five DM patients and five controls. The results from all patients demonstrated that major producers of TNFα were monocytes and mDCs (Figure 4). We observed a trend toward greater amounts of intracellular TNFα in monocytes from both DLE and DM patients compared to controls (*P *> 0.05) (Figure 4A). There was a statistically significant difference in TNFα intracellular staining of mDCs when both DLE (*P *< 0.001) and DM (*P *< 0.001) patients were compared to controls (Figure 4B).

### Mean fluorescence intensity of TNFα release by mDCs and monocytes

**Figure 5:**
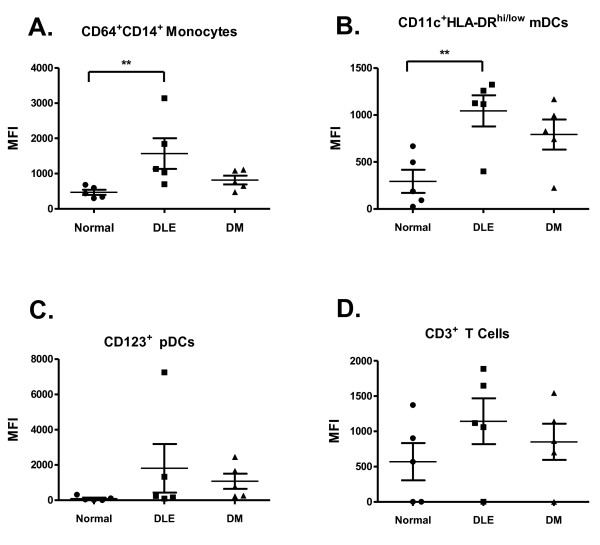
**Measurement of mean fluorescence intensity (MFI)**. The MFI of TNFα release from **(A) **CD64^+^CD14^+ ^monocytes, **(B) **CD11c^+^HLA-DR^hi/low ^myeloid dendritic cells, **(C) **CD123^+ ^plasmacytoid dendritic cells, and **(D) **CD3^+ ^T lymphocytes from five discoid lupus erythematosus (DLE) and five dermatomyositis (DM) patients, as well as from five controls, as determined by flow cytometry. Bars show the means and SD. ** *P *< 0.01 vs controls by Dunnett's multiple comparison test.

The mean fluorescence intensity (MFI) of TNFα release from CD64^+^CD14^+ ^monocytes and CD11c^+^HLA-DR^hi/low ^mDCs was measured among five DLE and five DM patients, as well as five controls. There was a 3.3-fold increase in the MFI of monocytes (*P *< 0.01) and a 3.6**-**fold increase in the MFI of mDCs (*P *< 0.01) from DLE patients compared to controls (Figure 5). There was no significant difference between the MFIs of both monocytes and mDCs from DM patients compared to controls.

### CD64^+^CD14^+ ^monocytes and CD11c^+^HLA-DR^hi/low ^myeloid dendritic cells were not significantly greater in DLE patients

**Figure 6:**
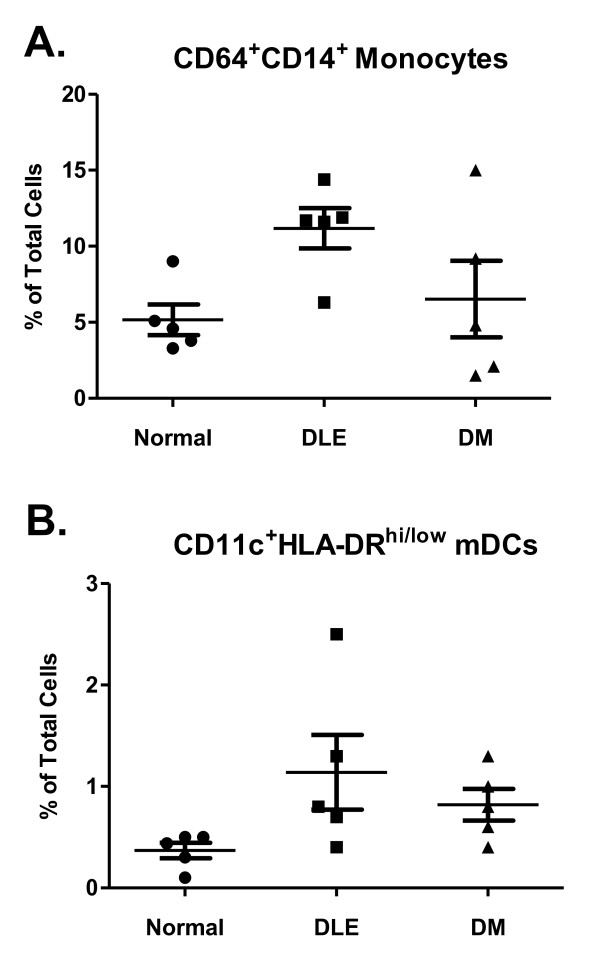
**Measurement of cell populations by flow cytometry**. **(A) **CD64^+^CD14^+ ^monocyte and **(B) **CD11c^+^HLA-DR^hi/low ^myeloid dendritic cell populations from five discoid lupus erythematosus (DLE) and five dermatomyositis (DM) patients, as well as from five controls, as determined by flow cytometry.

The size of the monocyte and mDC populations was measured in five DLE and five DM patients, as well as in five controls. The size of the CD64^+^CD14^+ ^monocyte population in DLE patients was 1.7-fold greater compared to DM patients (*P *> 0.05) and 2.2-fold greater compared to controls (*P *> 0.05) (Figure 6A). The size of the CD11c^+^HLA-DR^hi/low ^mDC population of DLE patients was 1.4-fold greater compared to that of DM patients (*P *> 0.05) and 3.1-fold greater than that of controls (*P *> 0.05) (Figure 6B).

## Discussion

The proinflammatory nature of TNFα in inflammatory diseases is well-established (reviewed in [7]). TNFα causes the upregulation of adhesion molecules and chemokines, which leads to attachment of inflammatory cells to vessels, rolling, emigration and eventually chemotaxis [24-26]. TNFα is an activating cytokine for fibroblasts and leukocytes, including dendritic cells. TNFα stimulation leads to increased production of IFN-γ, a cytokine that is also increased in lesional skin of DLE patients [12]. Our study demonstrates that TNFα release is significantly greater in PBMCs from DLE patients compared to controls.

We next determined which specific cell type was secreting the increased TNFα in DLE patients. Our flow cytometry results demonstrated that the majority of TNFα was produced by monocytes and mDCs, with a statistically significant difference between TNFα intracellular staining from mDCs of DLE patients (*P *< 0.001) and DM patients (*P *< 0.001) compared to controls. A significantly greater MFI from monocytes and mDCs from DLE patients compared to controls was also present, whereas there was no statistically significant difference in MFI from monocytes and mDCs between DM patients and controls. The significant elevation of the MFI in DLE patients compared to controls demonstrates that the monocytes and mDCs from DLE patients are synthesizing TNFα in greater quantities compared to controls. The MFI of the PBMCs from DLE patients is significantly greater than that of controls. Our ELISA results demonstrate an approximately 15-fold increase in TNFα release from DLE PBMCs compared to those seen in DM, and the flow cytometry results explain this finding in part. We realize the monocytes and mDCs comprise a relatively small percentage of circulating cells and that there may be another population of cells that we did not stain that could contribute to the increased TNFα release in DLE. We will investigate other cell types in the future.

We next explored whether there were any differences between the size of the monocyte and mDC populations among the DLE and DM patients and controls. We found a trend toward an increased population of monocytes in DLE patients compared to both DM patients and controls. The mDC population was trending toward greater numbers in DLE patients compared to controls. These flow cytometry results support our cultured PBMC ELISA results, which showed that there was a significantly greater amount of TNFα released from DLE patients. The increased amount of TNFα in monocytes and mDCs from DLE patients and the greater size of these cell populations in DLE patients may further explain why PBMCs from DLE patients release greater quantities of TNFα.

Plasmacytoid DCs have been implicated in the pathogenesis of both SLE and CLE. SLE is characterized by elevated levels of IFNα and IFNβ (both type I IFNs) [27,28]. Because pDCs are the main cell type producing IFNα and IFNβ, they have been implicated in disease pathogenesis. A higher proportion of pDCs are also present in the skin than in the blood of patients with SLE and CLE, suggesting that pDCs migrate from the circulation to the skin and may play a role in the production of skin lesions [29,30]. Our experiments demonstrate that TNFα levels produced by PBMCs vary with the different CLE subsets and with DM. We found that pDCs produced minimal amounts of TNFα compared to monocytes and mDCs. Therefore, we postulate that monocytes and mDCs may have a bigger role in the pathogenesis of DLE than was originally believed. The increased numbers of inflammatory cells seen in the skin of DLE patients relative to DM patients and controls may be explained, at least in part, by the increased TNFα produced in DLE, given the importance of TNFα in cell recruitment to endothelial cells and skin [25].

Flow cytometry showed that both CD11c^+^HLA-DR^hi ^and CD11c^+^HLA-DR^low ^mDCs produced TNFα and accounted for much of the TNFα production exhibited by PBMCs. Recently, an inflammatory mDC known as Tip-DC (TNFα-inducible nitric oxide synthase-producing dendritic cells) has been found in elevated amounts in psoriasis plaques compared with nonlesional skin from psoriasis patients and normal skin [9,31]. These Tip-DCs are CD11c^+^, BDCA-1^- ^and HLA-DR^+ ^[31]. Tip-DCs express HLA-DR at 10-fold lower levels compared to CD11c^+ ^and BDCA-1^+ ^mDCs. It is possible that some of the CD11c^+^HLA-DR^low ^mDCs in our DLE patients might actually have been Tip-DCs. It has been shown that bone marrow-derived monocytes accumulate and differentiate to Tip-DCs in the spleen, liver and lymph nodes of *Trypanosoma brucei brucei*-infected mice [32]. Another type of inflammatory mDCs, known as M-DC8^+ ^cells, has also recently been described that are CD11c^+^, CD14^+^, CD16^+^, CD64^- ^and HLA-DR^+ ^[33,34]. These M-DC8^+ ^cells are able to produce significantly greater amounts of TNFα in response to LPS compared to M-DC8^- ^monocytes and DCs [8,35]. Although we have not directly addressed these two populations of mDCs in our current studies, there is a possibility that these populations might contribute to enhanced TNFα production in DLE patients. Further studies designed to determine a potential role of these predominantly tissue-based DCs are being performed.

## Conclusions

Our findings suggest that DLE is associated with increased TNFα protein production from CD64^+^CD14^+ ^monocytes and CD11c^+^HLA-DR^hi/low ^mDCs. The MFIs of the monocyte and mDC populations are also increased in DLE, suggesting that these cells likely have a greater role in DLE than originally believed. Future studies using surface staining for Tip-DCs and M-DC8^+ ^mDCs by flow cytometry will allow us to explore the role of these cell populations in DLE.

## Abbreviations

CCLE: chronic cutaneous lupus erythematosus; CDASI: Cutaneous Dermatomyositis Area and Severity Index; CLASI: Cutaneous Lupus Erythematosus Disease Area and Severity Index; CLE: cutaneous lupus erythematosus; DLE: discoid lupus erythematosus; DM: dermatomyositis; ELISA: enzyme-linked immunosorbent assay; IFN: interferon; mDC: myeloid dendritic cell; PBMC: peripheral blood mononuclear cell; RT-PCR: real-time polymerase chain reaction; SCLE: subacute cutaneous lupus erythematosus; SLE: systemic lupus erythematosus; SLEDAI: Systemic Lupus Erythematosus Disease Activity Index; TLE: tumid lupus erythematosus; TNFα: tumor necrosis factor α.

## Competing interests

The authors declare that they have no competing interests.

## Authors' contributions

ASN and MMB carried out the PBMC cultures and TNF ELISA studies. AB and MW participated in the flow studies, and AB drafted the manuscript. VPW performed the CLASI assessments. VPW and ASN participated in the design of the study. MS performed the statistical analysis. VPW conceived the study, participated in its design and coordination and helped to draft the manuscript. All authors read and approved the final manuscript.

## References

[B1] Fabbri P Cardinali C Giomi B Caproni M Cutaneous lupus erythematosus: diagnosis and management Am J Clin Dermatol 2003 4 449 465 10.2165/00128071-200304070-00002 12814335

[B2] Gilliam JN Sontheimer RD Distinctive cutaneous subsets in the spectrum of lupus erythematosus J Am Acad Dermatol 1981 4 471 475 10.1016/S0190-9622(81)80261-7 7229150

[B3] Sontheimer RD The lexicon of cutaneous lupus erythematosus: a review and personal perspective on the nomenclature and classification of the cutaneous manifestations of lupus erythematosus Lupus 1997 6 84 95 10.1177/096120339700600203 9061656

[B4] Lin JH Dutz JP Sontheimer RD Werth VP Pathophysiology of cutaneous lupus erythematosus Clin Rev Allergy Immunol 2007 33 85 106 10.1007/s12016-007-0031-x 18094949

[B5] Caproni M Torchia D Cardinali C Volpi W Del Bianco E D'Agata A Fabbri P Infiltrating cells, related cytokines and chemokine receptors in lesional skin of patients with dermatomyositis Br J Dermatol 2004 151 784 791 [see comment] 10.1111/j.1365-2133.2004.06144.x 15491417

[B6] Smith ES Hallman JR DeLuca AM Goldenberg G Jorizzo JL Sangueza OP Dermatomyositis: a clinicopathological study of 40 patients Am J Dermatopathol 2009 31 61 67 10.1097/DAD.0b013e31818520e1 19155727

[B7] Bradley JR TNF-mediated inflammatory disease J Pathol 2008 214 149 160 10.1002/path.2287 18161752

[B8] Schäkel K Kannagi R Kniep B Goto Y Mitsuoka C Zwirner J Soruri A von Kietzell M Rieber E 6-Sulfo LacNAc, a novel carbohydrate modification of PSGL-1, defines an inflammatory type of human dendritic cells Immunity 2002 17 289 301 10.1016/S1074-7613(02)00393-X 12354382

[B9] Lowes MA Chamian F Abello MV Fuentes-Duculan J Lin SL Nussbaum R Novitskaya I Carbonaro H Cardinale I Kikuchi T Gilleaudeau P Sullivan-Whalen M Wittkowski KM Papp K Garovoy M Dummer W Steinman RM Krueger JG Increase in TNF-α and inducible nitric oxide synthase-expressing dendritic cells in psoriasis and reduction with efalizumab (anti-CD11a) Proc Natl Acad Sci USA 2005 102 19057 19062 10.1073/pnas.0509736102 16380428 PMC1323218

[B10] Black RA Rauch CT Kozlosky CJ Peschon JJ Slack JL Wolfson MF Castner BJ Stocking KL Reddy P Srinivasan S Nelson N Boiani N Schooley KA Gerhart M Davis R Fitzner JN Johnson RS Paxton RJ March CJ Cerretti DP A metalloproteinase disintegrin that releases tumour-necrosis factor-α from cells Nature 1997 385 729 733 10.1038/385729a0 9034190

[B11] Kawaguchi M Mitsuhashi Y Kondo S Overexpression of tumour necrosis factor-α-converting enzyme in psoriasis Br J Dermatol 2005 152 915 919 10.1111/j.1365-2133.2005.06440.x 15888146

[B12] Toro JR Finlay D Dou X Zheng SC LeBoit PE Connolly MK Detection of type 1 cytokines in discoid lupus erythematosus Arch Dermatol 2000 136 1497 1501 10.1001/archderm.136.12.1497 11115160

[B13] Popovic K Ek M Espinosa A Padyukov L Harris HE Wahren-Herlenius M Nyberg F Increased expression of the novel proinflammatory cytokine high mobility group box chromosomal protein 1 in skin lesions of patients with lupus erthematosus Arthr Rheum 2005 52 3639 3645 10.1002/art.21398 16255056

[B14] Drosera M Facchetti F Landolfo S Mondini M Nyberg F Parodi A Santoro A Zampieri S Doria A Role of soluble and cell surface molecules in the pathogenesis of autoimmune skin diseases Clin Exp Rheumatol 2006 24 1 Suppl 40 S7 S13 16466628

[B15] Zampieri S Alaibac M Laccarino L Rondinone R Ghirardello A Sarzi-Puttini P Doria A Peserico A TNF α is expressed in refractory skin lesions from patients with subacute cutaneous lupus erythematosus Ann Rheum Dis 2006 65 545 548 10.1136/ard.2005.039362 16096331 PMC1798098

[B16] De Bleecker JL Meire VI Declercq W Van Aken EH Immunolocalization of tumor necrosis factor-α and its receptors in inflammatory myopathies Neuromuscul Disord 1999 9 239 246 10.1016/S0960-8966(98)00126-6 10399751

[B17] Alecu M Coman G Alecu S Serological levels of apoptotic bodies, sFAS and TNF in lupus erythematosus Rom J Intern Med 2001 38-39 83 88 15529575

[B18] Sabry A Sheashaa H El-Husseini A Mahmoud K Eldahshan KF George SK Abdel-Khalek E El-Shafey EM Abo-Zenah H Proinflammatory cytokines (TNF-α and IL-6) in Egyptian patients with SLE: its correlation with disease activity Cytokine 2006 35 148 153 10.1016/j.cyto.2006.07.023 17027280

[B19] Maczynska I Millo B Ratajczak-Stefańska V Maleszka R Szych Z Kurpisz M Giedrys-Kalemba S Proinflammatory cytokine (IL-1β, IL-6, IL-12, IL-18 and TNF-α) levels in sera of patients with subacute cutaneous lupus erythematosus (SCLE) Immunol Lett 2006 102 79 82 10.1016/j.imlet.2005.08.001 16154204

[B20] Shimizu T Tomita Y Son K Nishinarita S Sawada S Horie T Elevation of serum soluble tumour necrosis factor receptors in patients with polymyositis and dermatomyositis Clin Rheum 2000 19 352 359 10.1007/s100670070027 11055823

[B21] Pachman LM Liotta-Davis MR Hong DK Kinsella TR Mendez EP Kinder JM Chen EH TNFα-308A allele in juvenile dermatomyositis: association with increased production of tumor necrosis factor α, disease duration, and pathologic calcifications Arthritis Rheum 2000 43 2368 2377 10.1002/1529-0131(200010)43:10<2368::AID-ANR26>3.0.CO;2-8 11037898

[B22] Krathen M Albrecht J Werth VP The cutaneous lupus disease activity and severity index as a validated outcome measure for cutaneous lupus erythematosus: comment on the article by Stamm et al Arthritis Rheum 2008 59 601 602 Comment on *Arthritis Rheum *2007, **57:**1287-1295 18383405 10.1002/art.23544 PMC3928015

[B23] Yassaee M Fiorentino D Okawa J Taylor L Coley C Troxel AB Werth VP Modification of the Cutaneous Dermatomyositis Disease Area and Severity Index, an outcome instrument Br J Dermatol 2010 162 669 673 10.1111/j.1365-2133.2009.09521.x 19863510 PMC2852630

[B24] Pober JS Bevilacqua MP Mendrick DL Lapierre LA Fiers W Gimbrone MA Jr Two distinct monokines, interleukin 1 and tumor necrosis factor, each independently induce biosynthesis and transient expression of the same antigen on the surface of cultured human vascular endothelial cells J Immunol 1986 136 1680 1687 3485132

[B25] Munro JM Pober JS Cotran RS Tumor necrosis factor and interferon-γ induce distinct patterns of endothelial activation and associated leukocyte accumulation in skin of *Papio anubis* Am J Pathol 1989 135 121 133 2505619 PMC1880213

[B26] Swerlick RA Lee KH Li KJ Sepp NT Caughman SW Lawley TJ Regulation of vascular cell adhesion molecule 1 on human dermal microvascular endothelial cells J Immunol 1992 149 698 705 1378077

[B27] Blanco P Palucka AK Gill M Pascual V Banchereau J Induction of dendritic cell differentiation by IFN-α in systemic lupus erythematosus Science 2001 294 1540 1543 10.1126/science.1064890 11711679

[B28] Kim T Kanayama Y Negoro N Okamura M Takeda T Inoue T Serum levels of interferons in patients with systemic lupus erythematosus Clin Exp Immunol 1987 70 562 569 2449306 PMC1542177

[B29] Johnson-Huang LM McNutt NS Krueger JG Lowes MA Cytokine-producing dendritic cells in the pathogenesis of inflammatory skin diseases J Clin Immunol 2009 29 247 256 10.1007/s10875-009-9278-8 19252974 PMC2874976

[B30] Farkas L Beiske K Lund-Johansen F Brandtzaeg P Jahnsen FL Plasmacytoid dendritic cells (natural interferon-α/β-producing cells) accumulate in cutaneous lupus erythematosus lesions Am J Pathol 2001 159 237 243 10.1016/S0002-9440(10)61689-6 11438470 PMC1850412

[B31] Zaba LC Fuentes-Duculan J Eungdamrong NJ Abello MV Novitskaya I Pierson KC Gonzalez J Krueger JG Lowes MA Psoriasis is characterized by accumulation of immunostimulatory and Th1/Th17 cell-polarizing myeloid dendritic cells J Invest Dermatol 2009 129 79 88 10.1038/jid.2008.194 18633443 PMC2701224

[B32] Guilliams M Movahedi K Bosschaerts T VandenDriessche T Chuah MK Hérin M Acosta-Sanchez A Ma L Moser M Van Ginderachter JA Van Ginderachter JA Brys L De Baetselier P Beschin A IL-10 dampens TNF/inducible nitric oxide synthase-producing dendritic cell-mediated pathogenicity during parasitic infection J Immunol 2009 182 1107 1118 19124754 10.4049/jimmunol.182.2.1107

[B33] Schäkel K Poppe C Mayer E Federle C Riethmüller G Rieber EP M-DC8+ leukocytes: a novel human dendritic cell population Pathobiology 1999 67 287 290 10.1159/000028081 10725804

[B34] de Baey A Mende I Riethmueller G Baeuerle PA Phenotype and function of human dendritic cells derived from M-DC8^+ ^monocytes Eur J Immunol 2001 31 1646 1655 10.1002/1521-4141(200106)31:6<1646::AID-IMMU1646>3.0.CO;2-X 11385608

[B35] de Baey A Mende I Baretton G Greiner A Hartl WH Baeuerle PA Diepolder HM A subset of human dendritic cells in the T cell area of mucosa-associated lymphoid tissue with a high potential to produce TNF-α J Immunol 2003 170 5089 5094 12734354 10.4049/jimmunol.170.10.5089

